# Preschool Children’s Memory for Word Forms Remains Stable Over Several Days, but Gradually Decreases after 6 Months

**DOI:** 10.3389/fpsyg.2016.01439

**Published:** 2016-09-27

**Authors:** Katherine R. Gordon, Karla K. McGregor, Brigitte Waldier, Maura K. Curran, Rebecca L. Gomez, Larissa K. Samuelson

**Affiliations:** ^1^DeLTA Center and Department of Communication Sciences and Disorders, University of Iowa, Iowa CityIA, USA; ^2^Department of Psychology, The University of Arizona, TucsonAZ, USA; ^3^School of Psychology, University of East AngliaNorwich, UK

**Keywords:** word learning, memory, retention, preschool children, word form

## Abstract

Research on word learning has focused on children’s ability to identify a target object when given the word form after a minimal number of exposures to novel word-object pairings. However, relatively little research has focused on children’s ability to retrieve the word form when given the target object. The exceptions involve asking children to recall and produce forms, and children typically perform near floor on these measures. In the current study, 3- to 5-year-old children were administered a novel test of word form that allowed for recognition memory and manual responses. Specifically, when asked to label a previously trained object, children were given three forms to choose from: the target, a minimally different form, and a maximally different form. Children demonstrated memory for word forms at three post-training delays: 10 mins (short-term), 2–3 days (long-term), and 6 months to 1 year (very long-term). However, children performed worse at the very long-term delay than the other time points, and the length of the very long-term delay was negatively related to performance. When in error, children were no more likely to select the minimally different form than the maximally different form at all time points. Overall, these results suggest that children remember word forms that are linked to objects over extended post-training intervals, but that their memory for the forms gradually decreases over time without further exposures. Furthermore, memory traces for word forms do not become less phonologically specific over time; rather children either identify the correct form, or they perform at chance.

## Introduction

In a typical word-learning task, children see an unknown object, either alone or with some familiar objects, and hear its name several times (e.g., see the dax). Afterward, they are presented with an array of objects and asked to point to the target when given the word form (e.g., Which one is the dax?) (see Carey, 2010; Swingley, 2010 for reviews). The majority of research on word learning has focused on these referent selection tests. However, word learning encompasses a variety of other abilities including the ability to retrieve the word form when given the target referent (i.e., form tests). Although far less commonly used than referent tests, typical form tests include asking children to name a trained object (e.g., What is this one called?) (see Dollaghan, 1985; Gray, 2003; Booth et al., 2008; Hoover et al., 2010 for examples). When tested immediately after training of word-referent pairs, children perform well on referent tests but poorly on form tests (Kiernan and Gray, 1998; Gray, 2003; Booth et al., 2008; Munro et al., 2012). In fact, performance on form tests that require naming is often at floor; clearly such tests are not sensitive to children’s word learning. Critically, given the relatively infrequent use of form tests, and the lack of sensitively of these tests, our understanding of children’s ability to encode and retrieve forms is limited. An additional gap in the literature is that children’s memory for words is typically only assessed immediately after training. Thus, we lack a detailed understanding of how children’s memory for word-referent pairs, particularly their memory for forms, changes across post-training delays. A related unexplored question is how children’s phonological representations of forms change across post-training delays. In the current work, we begin to address these gaps by assessing 3- to 5-year-old children’s memory for forms across short and long delays using a more sensitive word form test.

### Measures of Word Form

Children typically perform much better in referent than form tests when tested immediately after training, yet these tests differ in task demands. Referent tests allow for manual responses and recognition memory in that children respond by pointing to one of the objects presented to them. In contrast, form tests require verbal responses and recall memory in that children must produce forms and are not given a variety of forms to choose from. To address the difference between test outcomes, Gordon and McGregor (2014), following Storkel (2001) and Nash and Donaldson (2005), developed a form test with task demands similar to referent tests. In this test, which we will call the dot test, the trained target object and a paper with three large dots on it are placed in front of the child. The experimenter gives the child three forms to choose from by pointing to one of the dots while producing each form. The tested forms are the target; a minimally different form that differs from the target in either the initial, medial, or final constant; and a maximally different form that differs from the target in number of syllables and the majority of phonemes represented. For example, if the trained form was “dorb,” the experimenter would ask, “What is this one called?” while pointing to the target object: “Is it a dorb?” while pointing to the first dot; “Is it a vorb?” while pointing to the second dot; “Or is it a zinnip?” while pointing to the third dot. Children respond by pointing to the dot that corresponds with one of the forms, stating a form, or doing both.

In Gordon and McGregor (2014), 4- to 6-year-old children received five exposures each to 12 word-object links. A week later, children’s memory for the links was tested through the dot test and a traditional referent test. Children’s performance on the dot test was well above chance, much better than performance on typical form tests that are administered immediately after training. Given that the dot test is a more sensitive measure of children’s memory for forms than typical tests, we can utilize this test to assess children’s ability to retrieve word forms across various delays.

### Long-Term Memory for Word Forms

The majority of research on children’s word learning has focused on assessing their memory for word-referent links immediately after training. Although less common, there is some work investigating children’s memory for words across various post-training delays. For example, after a minimal number of exposures, 2-year-old children are good at selecting the referent immediately after training, yet they are poor after delays as short as 5 mins (Horst and Samuelson, 2008). In contrast, preschool-age children can maintain a word over a delay of several days (Rice et al., 1994), 1 week (Carey and Bartlett, 1978; Markson and Bloom, 1997; Waxman and Booth, 2000; Holland et al., 2015), 1 month (Markson and Bloom, 1997), and several months (Kan, 2014) when their memory is assessed through referent tests. These differences are consistent with broader work on children’s memory development, namely the length of time that children can retain a memory is positively correlated with age (see Bauer, 2015a).

Although assessing children’s long-term retention of word-referent links through referent tests is rare in the literature, assessing children’s retention of word forms over delays is even rarer. This is likely due, in part, to a lack of sensitive measures for children’s memory of forms. For example, Kan and Kohnert (2012) and Kan (2014) assessed bilingual preschool children’s memory for words through both referent and form tests immediately after training and after a 1-week and 4-month delay. As children performed near floor in the form test at all time points, not much information was gathered on children’s memory for forms. Munro et al. (2012) provide more information about children’s long-term memory for forms. In this study, 2- to 3-year-old children were given six exposures to novel word-referent pairs then tested 1 min, 5 mins, and several days later. At each test point, they were first asked, “What is this one called?”, but were given the first syllable of the target word as a memory cue if they did not produce a form. The researchers found that children’s memory for the forms sharply decreased during the 5-min interval, but did not differ between tests given 5 mins and several days after training. Some children who were unable to demonstrate memory for the forms during free recall were able to do so after cuing. However, their performance remained near floor. After several days children produced, on average, 4% of the forms through free recall, and 7% of the forms after cueing.

Do children remember more about newly learned word forms than production tests-even cued production tests-reveal? Recognition/manual versions of form tests, such as the dot test, offer a more sensitive measure to address this question. In addition to addressing this primary question, the influence of factors both internal and external to the child on long-term retention can be explored through this more sensitive measure. An internal factor that may affect children’s ability to encode and retrieve forms is their current vocabulary level. Research that has explored the relationship between vocabulary and word learning, as measured by referent tests, has yielded mixed results; some researchers have found evidence of a relationship (Gathercole et al., 1997; Masoura and Gathercole, 2005; Munro et al., 2012; Law and Edwards, 2015) and others have not (Gray, 2003; Tan and Schafer, 2005). These mixed results are likely due to variations in the child’s age, cognitive abilities, and specific demands of the word-learning task.

Critically, although research with infants has shown that vocabulary size is related to their ability to learn word forms that are linked to objects (Werker et al., 2002), little work has explored this question in older children. Furthermore, the question of how vocabulary is related to children’s ability to maintain trained words across delays remains largely unexplored. Munro et al. (2012) is an exception that addresses both of these limitations in that they found a relationship between 2- and 3-year-old children’s receptive vocabulary and their ability to retrieve trained words after a 1-min, 5-min, and several-day delay. However, we are unaware of any work that has looked at how vocabulary size is related to children’s ability to retain words across delays longer than a week.

An external factor that has been shown to affect retrieval is context cues, such as the similarity of the décor and location of the room where learning and testing occurs, or whether the same person administers the training and test (Smith and Vela, 2001; Hupbach et al., 2008; Hupbach et al., 2011; Vlach and Sandhofer, 2011; Goldenberg and Sandhofer, 2013). There is some work demonstrating that consistency of context cues positively influences children’s ability to learn (Horst et al., 2011), retrieve (Goldenberg and Sandhofer, 2013), and generalize (Vlach and Sandhofer, 2011) novel word-referent pairs (also see Horst, 2013). One critical finding in the adult literature is that the longer the delay, the more likely context cues are to affect retrieval (Smith and Vela, 2001). However, currently, it is not understood how context cues affect preschool-age children’s ability to retrieve word-referent links after longer delays.

### Phonological Specificity of Word Forms

In addition to providing more information about children’s memory for forms across delays, the dot test can offer new information about how the phonological specificity of children’s representations of forms changes post-training. Phonological specificity is typically assessed by measuring children’s tendency to select or visually focus on the target object when they are given correct pronunciations vs. mispronunciations of the target form (see Vihman, 2014 for a review). The vast majority of this work has focused on infants ages 2 years or younger. Children’s responses vary with age (Vihman, 2014), vocabulary level (Werker et al., 2002), manner and degree of phonological variation from the target (Halle and de Boysson-Bardies, 1996), familiarity with the form (Halle and de Boysson-Bardies, 1994, 1996; Fennell and Werker, 2003; Ballem and Plunkett, 2005), sentence context (Cole and Perfetti, 1980), language (Vihman et al., 2004), and number of exposures to the form (Kay-Raining Bird and Chapman, 1998). Despite this variation, the general conclusion is that children encode fairly specific representations of forms after training, but that performance varies with the particulars of the task.

Because children are selecting among objects in these tests, the familiarity and similarity of objects, and whether objects were previously named can affect performance (Oviatt, 1980; Horst et al., 2011; Kucker and Samuelson, 2012). This limitation has been addressed in two ways. First, researchers have assessed children’s memory for forms in the absence of an object, which does improve performance (see Werker et al., 2002 for a review). However, a limitation of this methodology is that it is testing children’s recognition of the trained form, not their recognition of a specific form linked to a specific object. Second, researchers have assessed children’s representations of word forms through form tests (i.e., asking children “What is this one called?”), which eliminate the need to select among objects. A limitation of this method is its heavy recall demands without a supportive cue, which contributes to near floor performance. Additionally, when children do attempt the forms, it is unclear whether mispronunciations reflect children’s phonological representations or their production difficulties (Swingley and Aslin, 2000).

Although not broadly used, there are two innovative methodo logies to measure children’s phonological representations of forms that do not require verbal productions or object selection. Namely, children are asked to indicate through yes/no responses whether individual forms apply to a specific object, or they are asked to identify the correct form for an object when there are several forms to choose from, similar to the dot test (see Weismer and Hesketh, 1996, 1998; Alt et al., 2004; Alt and Plante, 2006). Similar to measures typically used with very young children, these assessments reveal that preschool- and school-age children encode fairly specific representations of forms when assessed immediately after training.

Given that the dot test includes a target and a foil that comprise a minimal pair, it can reveal how the specificity of forms changes across various post-training intervals. One possibility is that with adequate memory supports during training, the specificity of encoded forms will remain stable over longer delays. Vlach and Sandhofer (2012) found that children could recognize the target object when given the form (i.e., a referent test) over the delay of a month when given adequate memory supports during training. Thus, given adequate memory supports, children may maintain the ability to retrieve the form via a form test after a longer delay. A second possibility is that forms will become more specific over time due to consolidation. There is some evidence that this happens over shorter time scales (e.g., 24 hrs) (Henderson et al., 2012). However, research on forgetting curves suggest that this is unlikely to happen over longer time scales without further training (Henderson et al., 2012; Murre and Dros, 2015). A third possibility is that phonological representations will become weaker over time. It is possible that it is harder to retain the ability to identify the form when given the target object than the reverse. Thus, the ability to recognize forms may show a typical forgetting curve over time even when given memory supports during training.

If representations of forms become weaker over time, the dot test could offer insights into how they change. If the representations become less phonologically specific, children should gradually decrease selections of the target form while increasing selections of the minimally different form. However, children’s representations of forms could remain fairly specific, but children will lose the ability to retrieve the memory trace or the memory trace could disappear completely. In this case, when children no longer reliably select the target form, they should be at chance responding when choosing between the target, a minimally different form, and a maximally different form. Gordon and McGregor (2014) found that children were no more likely to select the minimally different form than the maximally different form 1 week after training; providing support for the second hypothesis. However, as children were not tested directly after training, or at time points longer than a week, it is unknown how the phonological memory for forms changes across various delays.

### The Current Study

The primary questions of the current study are twofold: (1) How does the number of word forms that children correctly identify change based on the length of the delay?, and (2) How do children’s phonological representations of word forms change based on the length of the delay? To address these questions, in Experiment 1 we trained children on word-object links in a similar manner to Gordon and McGregor and assessed children’s memory for the forms 10 mins after training (short-term delay) and 2 to 3 days after training (long-term delay). For Experiment 2, a subset of children who participated in Experiment 1 were tested on their retention of forms 6 months to 1 year after training (very long-term delay). Using the dot test as the assessment measure at each time point provided insight into whether phonological representations become less specific over time. Finally, to address the question of whether children’s selections in the dot test simply reflect their ability to identify a form they heard during training, or their ability to identify the specific form that goes with a specific object, we administered a 4-dot version of the test after the final testing session. In this test, children are given the target form for the object being asked about and a minimal pair of the target, plus an alternate trained form that is linked to another object and a minimal pair of that form. We report this methodology and results as Experiment 3.

As we wanted to focus on differences in retrieval at various time points, we decided to maximize encoding by including optimal memory supports during training. Thus, training included: multiple spaced presentations of word-referent links (McGregor et al., 2007; Vlach et al., 2008), ostensive naming (Horst and Samuelson, 2008; Axelsson et al., 2012), and the opportunity to handle and manipulate the objects (Scofield et al., 2009).

We also investigated how individual and contextual factors affected performance after various delays. Thus, we assessed children’s vocabulary level and general language comprehension and production abilities. Additionally, to explore whether younger children reliably show retrieval of forms after various delays and how retrieval of forms varies between age groups, we assessed 3-year-old children’s in addition to 4- and 5-year-old children’s performance on the dot test. With regards to environmental factors, we assessed the effect of room environment (i.e., location and décor) and experimenter on children’s ability to retrieve previously learned word-object links.

Based on past findings, we predicted that given memory supports during training, children would maintain a reliable memory for forms over shorter (2 to 3 days) and longer (6 months to 1 year) delays. Additionally, children should maintain fairly specific representations of forms across short retention intervals. However, given the lack of research we do not make a firm prediction about how phonological representations change across longer post-training delays. Age and language abilities should be positively related to children’s ability to encode and retain forms. Furthermore, context cues should aid retrieval after longer delays, but are less likely to do so after shorter delays.

## Experiment 1

### Materials and Methods

#### Participants

Participants included sixteen 3-year-old children (mean age = 41.13 months, range = 36–46 months, females = 9) and sixteen 4- to 5-year-old children (mean age = 58 months, range = 49–67 months, females = 8). According to parental report, none of the children had a history of speech or language problems. Children were recruited via mass email to university faculty, staff, and students. The data from five additional participants were excluded: two due to experimenter error and three because the children refused to continue.

According to self-report, mothers of the participants completed a mean of 18.11, *SD* = 3.04, years of education (one participant did not provide this information). Fathers of the participants completed a mean of 18.31, *SD* = 3.60, years of education (two participants did not provide this information). Children’s racial/ethnic backgrounds were as follows: white/non-Hispanic = 22, white/Hispanic = 2, Hispanic (without race provided) = 2, black/non-Hispanic = 2, biracial = 2, not available = 2.

All reported experiments received approval from the Institutional Review Board of the university of the lead author. Parents and/or guardians of all participants gave written informed consent for their child to participate.

#### Stimuli

The objects presented to the children were similar to the objects in Gordon and McGregor (2014). There were 12 referent categories: each included 1 referent exemplar (the prototype) and 1 referent exemplar that varied from the other one in color, size, or both (the non-prototype). Two referent exemplars for each category were used during training as some similarity and some variation within categories promotes both learning and generalization (Rost, 2011; Twomey et al., 2013). There was one generalization exemplar for each referent category used during testing that varied from the other two exemplars in color, size, or both. Stimuli also consisted of 12 foil categories that each included 1 foil exemplar (the prototype) and 1 foil exemplar that varied from the other one in color, size, or both (the non-prototype).

A novel word was assigned to each of the 12 referent categories. Six were monosyllabic and six were disyllabic; all had low lexical neighborhood densities and similar phonotactic probabilities. The words were designed to maximize learning in that they were composed of early acquired sounds, and none shared the same initial syllable (Creel et al., 2006). The words were divided into two sets that each contained 3 monosyllablic and 3 disyllabic words. Place and manner characteristics varied within each word set, but the two sets were similar in place and manner features represented. Twenty-four additional novel words served as foils in the dot test. Twelve foils, the minimally different forms, varied from the targets in either onset, medial or final consonant with the position of change counterbalanced across foils. The other 12 foils, the maximally different forms, varied from the target in number of syllables and in the majority of phonemes.

#### Procedure

The majority of children passed a pure-tone audiometric screening administered in a non-soundproofed laboratory room at 1, 2, and 4 kHz at 20 dB and 0.5 kHz at 25 dB. Two children responded at 30 dB in the right ear and 25 dB in the left ear at 0.5 and 4 kHz, respectively. One child responded at 30 dB in the right ear and 40 dB in the left ear at 0.5 kHz. As all three children passed at 25 dB at all other levels, their data were retained.

Children participated for two consecutive weeks. Each week included one training session and one testing session conducted 48–72 hrs later. The first training session occurred in a room on the lowest floor of a building on campus that was decorated like a living room with a couch and a colorful rug. The second training session occurred in a room on the top floor of the same building decorated like an office with two chairs on either side of a desk and a gray rug. Each testing session was conducted in the training context used that week or in a novel testing context (order counterbalanced across children). The novel testing context was a room that resembled a hospital room in the building next door.

During training sessions, the experimenter presented the objects from one of the sets on a tray on her lap while she sat on the couch or presented the objects on the desk. Set order was counterbalanced across children. Children were shown a prototype referent exemplar (i.e., the red dorb) that was named two times (i.e., Look at this dorb. See a dorb), followed by a prototype foil that was not named (i.e., Look at this. See this.), until all 6 referent exemplars and 6 referent foils had been presented. Children were encouraged to handle the objects. As spacing of presentations has been shown to aid retention, children were given a 3-min stretch break after all the objects were presented once (Vlach et al., 2008). After the first break, the experimenter presented a non-prototype referent exemplar (e.g., the blue dorb) that was named (i.e., Look at this dorb), followed by a non-prototype foil (i.e., Look at this) until all 6 target and 6 foil categories had been shown, followed by a 10-min break that involved riding the elevator in the building. After the second break, children were shown the prototype referent exemplar again (e.g., the red dorb) named one time (i.e., We’ve got this dorb), and then shown the prototype foil (We’ve got this). After the prototype exemplar from all the referent categories and foil object categories had been shown a second time, the child took another break on the elevator before the short-term test. The unnamed foils were presented during training so that the training protocol would match previous work, but they were not used during testing in the current experiment.

For the short-term test, the experimenter placed a paper with three large dots on it on the tray or desk. The experimenter showed a prototype referent exemplar (e.g., the red dorb) and asked the child, “What is this one called?” The experimenter than produced three forms (e.g., the target, minimally different form, and maximally different form), and pointed to one of the dots on the paper as she produced each form. The order in which the three choices were produced was counterbalanced across test trials. Children could state a form, point to a dot, or do both. If the child pointed to a dot and stated a form that did not match that dot, the stated form took precedence. If a child stated a form that was not exactly like one of the options given, the child was asked to repeat his/her answer and the response was coded as the form that it was most similar to. To challenge children’s memory, the referent exemplars were tested in a different order than they were presented during training. Namely, the 2nd, 4th, and 6th referent exemplars trained were tested first, followed by the 1st, 3rd, and 5th exemplars. Children received two practice trials before the short-term test that included familiar objects (i.e., a book and a car) and were given three forms to choose from (e.g., “What is this one called? Is this a car, a lar, or a daxim?”).

Two to three days later, children’s long-term memory of forms was tested using the dot test and generalization exemplars of each referent category. Generalization exemplars were used to sufficiently challenge children’s long-term memory such that any context effects would be revealed. The objects were tested in the same order as the short-term test (e.g., 2nd, 4th, 6th, 1st, 3rd, 5th). However, the order of the presentation of the forms (target, minimally different form, maximally different form) varied between the short- and long-term tests.

When tested in the familiar context, at the end of the session children were brought outside of the room and the door was closed. The children were asked, “Do you remember the room that we were just in? What did it look like?” They were shown a paper with photos of four versions of the room: the same décor and the same arrangement of furniture, different décor and the same arrangement of furniture, the same décor and a different arrangement of furniture, and different décor and a different arrangement of furniture than the actual room. When tested in the novel context, they were shown the four photos at the end of testing and asked, “Do you remember the room where we first played with these things? What did it look like?”

Children also completed a variety of standardized tests: a receptive vocabulary test, PPVT-4 (Dunn and Dunn, 2007), and the comprehension and expression tests from the Preschool Language Scale, PLS-4 (Zimmerman et al., 2002). All standardized testing was conducted while seated on the rug of each room to encourage the specific training context (i.e., on the couch or at the desk) to be uniquely associated with the object sets.

### Results

A series of one-sample *t*-tests was conducted to compare the number of times children selected the target form to chance (2 correct responses out of 6 3AFC-alternative forced choice trials). Because we performed four *t*-tests for each age group, a Bonferroni correction was used to determine significance, in this case 0.05/4 = 0.0125. Both age groups performed above chance at all time points (see **Table 1**). A t-test was conducted to assess whether performance at the long-term test differed from performance at the short-term test. Children’s performance did not significantly differ at these two times points *t*(31) = 0.474, Cohen’s *d* = 0.128.

**Table 1:** Comparisons of children’s performance at each time point in Experiment 1 to chance.

		Short-Term, 10 mins	Long-term, 2–3 days
3-year-olds	Week 1	Mean = 4.125 (1.821), *t*(15) = 4.667, *p* < 0.0001, Cohen’s *d* = 1.217	Mean = 4.000 (1.673), *t*(15) = 4.204, *p* = 0.001, Cohen’s *d* = 0.998
	Week 2	Mean = 2.812 (1.328), *t*(15) = 2.908, *p* = 0.011, Cohen’s *d* = 0.951	Mean = 3.063 (1.340), *t*(15) = 3.171, *p* = 0.006, Cohen’s *d* = 0.682
4- and 5-year-olds	Week 1	Mean = 5.125 (0.806), *t*(15) = 13.190, *p* < 0.0001, Cohen’s *d* = 3.298	Mean = 5.063 (1.237), *t*(15) = 9.906, *p* < 0.0001, Cohen’s *d* = 2.478
	Week 2	Mean = 3.688 (1.621), *t*(15) = 4.163, *p* = 0.001, Cohen’s *d* = 1.041	Mean = 3.313 (1.580), *t*(15) = 3.323, *p* = 0.005, Cohen’s *d* = 0.831


To assess the effects of the various factors on short- and long-term memory for forms, a mixed effects logistic regression in an R environment, using the lme4 package was conducted. Models predicted log odds of a correct response on the dot test. See **Table 2** for the predictors that were explored for the short-term and long-term test. Age was coded categorically as “Three” or “Four/Five” on the basis of age at the initial testing session, and was included as a covariate in all models.

**Table 2:** Covariates tested for each model.

Predictor	Results analyzed for
Set (A/B)	All
Order (Week 1/Week 2)	All
Age	All
PPVT-IV raw score	All
PLS-4 expressive raw score	All
PLS-4 comprehension raw score	All
Context (Familiar/Novel room)	Long-term
Context (Familiar/Novel building, room, and experimenter)	Very long-term
Accuracy: short-term test	Long-term, very long-term
Accuracy: long-term test	Very long-term
Delay in days (Standardized)	Very long-term


For the model looking at short-term performance, the maximal random effects structure supported by the data included random intercepts for participant and item. Preliminary testing found no reliable differences in performance associated with object set A or B (*p* > 0.10). Model fit did not drop when omitting this predictor. Thus, all data were collapsed across training set for the remainder of the analyses.

The final model included main effects for week and age (see **Table 3**). A significant effect of week emerged, such that log odds of correct performance was lower during week 2 than week 1 (*z* = -4.93, *p* < 0.01). A reliable main effect of age emerged, such that log odds of a correct response were higher for the 4- and 5-year-old group than the 3-year-old group (*z* = 1.99, *p* < 0.05). There were no significant effects of PLS comprehension raw score (*z* = 0.73), PLS expressive raw score (*z* = -0.38) or PPVT-IV raw score (*z* = -0.43).

**Table 3:** Final model for the short-term test.

	Estimate	Standard error	*z* value	Pr(>|z|)
Intercept	2.36	0.51	4.63	<0.01
Week^a^	-1.25	0.25	-4.93	<0.01
Age^b^	0.78	0.39	2.00	0.05


*^a^reference group is Week 1; ^b^reference group is 3-year-olds.*

For the model looking at long-term performance, the maximal random effects structure supported by the data included random intercepts for participant and item. We systematically tested the same predictors as those included in the short-term test model with two additions: testing context (familiar or novel testing room) and accuracy at the short-term test. Preliminary testing found no reliable differences in performance associated with object set or context of the test (all *p* > 0.10). Model fit did not drop when omitting these predictors. Thus, all data were collapsed across training set and context for the remainder of the analyses.

**FIGURE 1:**
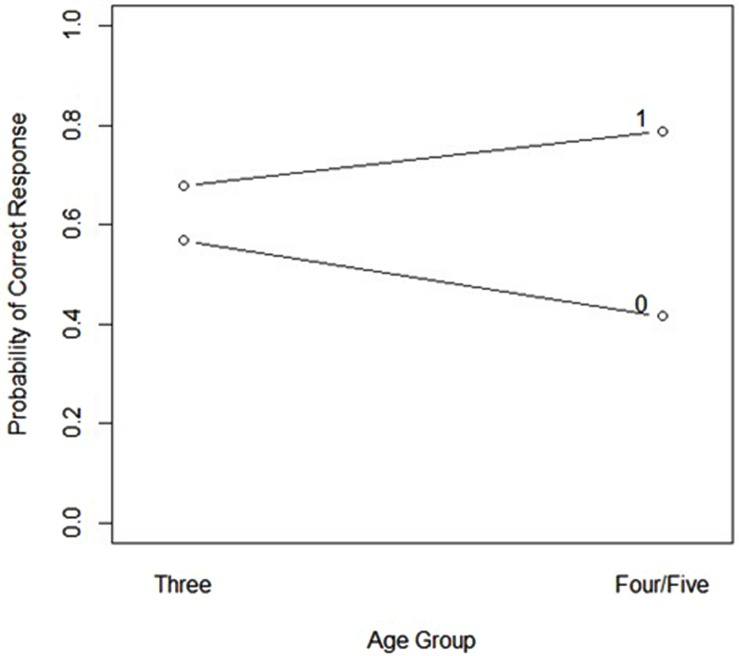
**Likelihood of a correct response on the long-term test plotted by response accuracy on the short-term test and age, short-term accuracy is coded as 0 (incorrect) or 1 (correct) above**.

The final model included main effects for week, age, PPVT-IV raw score, and short-term accuracy; and an interaction between age and short-term accuracy (see **Table 4**). A significant main effect of week emerged, such that log odds of a correct response were lower for items taught during the second week (*z* = -3.78, *p* < 0.01). Also, a reliable, positive, main effect of PPVT-IV raw score emerged, such that log odds of a higher PPVT score predicted more accurate responses at the long-term test (*z* = 2.04, *p* < 0.04). No significant main effect of PLS Comprehension or Expressive scores emerged (*z* = 1.47, n.s. and *z* = 0.65, n.s. respectively). A significant interaction between age and prior accuracy emerged (*z* = 2.22, *p* = 0.03). Three-year-olds tended to show little effect of prior accuracy on a specific item (e.g., dorb) on performance of that item at the long-term test. The 4- and 5-year-old participants demonstrated increased log odds of a correct response at long-term in association with a correct short-term response, and decreased log odds of a correct response at long-term in association with an incorrect short-term response (see **Figure 1**).

**Table 4:** Final model for the long-term test.

	Estimate	Standard error	*z* value	Pr(>|z|)
Intercept	0.80	0.66	1.22	0.22
Week^a^	-0.97	0.26	-3.78	<0.01
PPVT-IV raw score	0.01	0.01	2.04	0.04
Age^b^	-0.61	0.51	-1.21	0.23
Short-term response^c^	0.47	0.36	1.32	0.19
Age ^∗^ Short-term response	1.18	0.53	2.22	0.03


*^a^reference group is Week 1; ^b^reference group is 3-year-olds; ^c^reference group is incorrect.*

#### Response Type

Four coders rated whether children said a form, pointed to a dot, or did both for each response in the tests (regardless of whether the child indicated the correct form or not). At least two coders independently rated each video and inter-rater reliability was 99%. In cases where there was a disagreement, the primary investigator watched the video to resolve the discrepancy. Five children’s data were excluded from this analysis because videos of the sessions did not record successfully. Thus, the following analyses included thirteen 3-year-old children, and ten 4- and 5-year-old children. There was one trial in which one 3-year-old refused to respond, but his responses to all other trials were included.

Three-year-old children: out of all 312 trials (14 children, 24 trials each), children just pointed to a dot for 131 or 42% of the trials (mean 10.07 trials per child, *SD* = 9.10), just stated a form for 97 or 31% of the trials (mean 7.46 trials per child, *SD* = 7.36), and both pointed to a dot and stated a form for 83 or 27% of the trials (mean 6.38 trials per child, *SD* = 6.48). Four- and 5-year-old children: out of all 240 trials (10 children, 24 trials each) children just pointed to a dot for 118 or 49% of the trials (mean 11.80 trials per child, *SD* = 9.03), just stated a form for 82 or 34% of the trials (mean 8.2 trials per child, *SD* = 9.84), and both pointed to a dot and stated a form for 40 or 17% of the trials (mean 4.00 trials per child, *SD* = 5.14).

Children were fairly consistent in the type of responses they gave. For the 3-year-olds, children used the same response type an average of 72% (range 37.5–100%) of the time. Five children pointed most often, five children said a form most often, and three children did both most often. For the 4- and 5-year-olds, children used the same response type an average of 79% of the time (45.8–100%). Five children pointed most often, three children said a form most often, and two children did both most often.

To assess whether type of response (i.e., point, say, both) was related to accuracy on short- and long-term memory tests for forms, a mixed effects logistic regression in an R environment, using the lme4 package, was conducted. Random effects for participant and item were included. Age was coded categorically as “Three” or “Four/Five” on the basis of age at the initial testing session, and was included as a covariate. This analysis revealed no main effect for type of response (*p* > 0.10).

#### Word-Level Factors

Only the number of choices of the target form has been analyzed thus far. To compare children’s choices of the target, minimally different form, and maximally different forms at the short- and long-term tests, we conducted a series of *t*-tests (Bonferroni correction, 0.05/6 = 0.0083). At both time points, children were more likely to select the target than the minimally different form [short-term *t*(31) = 8.330, *p* < 0.0001, Cohen’s *d* = 4.253, long-term *t*(31) = 7.715, *p* < 0.0001, Cohen’s *d* = 3.942] and more likely to select the target than the maximally different form [short-term *t*(31) = 8.580, *p* < 0.0001, Cohen’s *d* = 4.027, long-term *t*(31) = 8.091, *p* < 0.0001, Cohen’s *d* = 3.849], but the number of selections of the minimally different and maximally different forms did not differ [short-term *t*(31) = 0.084, *p* = 0.934, Cohen’s *d* = 0.015, long-term *t* (31) = 0.685, *p* = 0.498, Cohen’s *d* = 0.383].

Children’s selection of the target, the minimally different form, and the maximally different form were also compared across the short- and long-term test (Bonferroni correction, 0.05/3 = 0.016). Children’s selections did not differ across the two tests: target, *t*(31) = 0.725, *p* = 0.474, Cohen’s *d* = 0.128; minimally different *t*(31) = 1.052, *p* = 0.301, Cohen’s *d* = 0.186; maximally different *t*(31) = 0.109, *p* = 0.914, Cohen’s *d* = 0.019.

We analyzed whether children were more likely to select the minimally different form based on the position of change (initial, medial, final) from the target based on their combined responses to the short- and long-term test. Thus, we conducted a repeated measures ANOVA with age (3-year-old, 4- and 5-year-old) as the between-subjects factor and position of change (initial, medial, and final) as the within-subjects factor, and the number of selections of the minimally different form as the dependent variable. There was not a significant main effect for position of change, *F*(2,29) = 2.967, *p* = 0.067, $\eta_{p}^{2}$ = 0.170 or age *F*(2,30) = 3.946, *p* = 0.056, $\eta_{p}^{2}$ = 0.116, and there was not a significant age by position of change interaction *F*(2,29) = 0.819, *p* = 0.451, $\eta_{p}^{2}$ = 0.053.

The number of one-syllable words and the number of two-syllable words that children correctly identified across the short- and long-term test were compared through a mixed-model ANOVA with age (3-year-olds, 4- and 5-year-olds) as the between-subjects factor and number of syllables (one, two) as the within-subjects factor. Children correctly identified more two-syllable words than one-syllable words *F*(1,30) = 4.416, *p* = 0.044, $\eta_{p}^{2}$ = 0.128, but there was no age by number of syllables interaction.

#### Memory for Context

One child’s data was excluded from the results of this test, as he was not shown the photos of the rooms after being tested in the familiar room. Chi-square analyses revealed that there was not a significant relationship between photo selection and age group χ(3) = 5.560, *p* = 0.135, nor a significant relationship between photo selection and the novel or familiar testing condition χ(3) = 3.159, *p* = 0.368. Thus, all children’s responses in both the novel and familiar context were analyzed together. Children’s selections of the training room at long-term test were compared to chance through a series of *t*-tests (Bonferroni Correction, 0.05/4 = 0.0125): children selected the correct photo significantly above chance *t*(61) = 4.159, *p <* 0.0001, Cohen’s *d* = 0.528. Their selections of the photo with the same décor, but a different arrangement, *t*(61) = 1.484, *p* = 0.653, Cohen’s *d* = 0.057, and the photo with different décor and the same arrangement *t*(30) = 0.177, *p* = 0.143, Cohen’s *d* = 0.188 did not differ from chance. Their selections of the photo with different décor and a different arrangement *t*(30) = 4.858, *p* < 0.0001, Cohen’s *d* = 0.617 were significantly below chance.

### Summary of Results

In responding to the dot test, both younger (3-year-olds) and older (4- and 5-year-olds) children demonstrated a memory for forms 10 mins and 2 to 3 days after training, and their performance at these two time points did not differ. This finding is in marked contrast to past results in which children performed near floor on form tests when they were asked to recall and produce the forms immediately after training. Age and week of training were both shown to be reliably related to performance at the short- and long-term test with older children performing better than younger children and children performing better during week 1 than week 2. Additionally, for older children, performance on a specific item at the short-term test was related to performance on that item at the long-term test, but this was not true of younger children. With regards to the specificity of the representation of word forms, children selected the target form reliably more than the other two forms at both the short- and the long-term test, and the number of selections of the target form did not differ based on delay.

## Experiment 2

There were two main purposes for Experiment 2: (1) To assess whether children could retrieve forms months after training (very long-term delay test), and whether their short- and long-term performance was related to their very long-term performance, and (2) To assess how delay length was related to children’s ability to retrieve the target forms. Additionally, to tap memory traces not detectable via the dot test, we retrained children on the word-object links and retested them. This allowed us to assess whether any residual memory trace would aid in relearning the forms.

### Materials and Methods

#### Participants

Nineteen of the 32 children returned for a long-term follow up (female, *n* = 11). Twelve of the returning children were 3-years-old and 7 were 4- to 5-years-old during initial training. The racial/ethnic backgrounds of the children were: white/non-Hispanic = 13, white/Hispanic = 2, Hispanic (without race provided) = 2, black/non-Hispanic = 2, biracial = 2, not available = 2. Age of original testing, maternal education in years, paternal education in years, raw PPVT score, raw PLS comprehension and expression scores, and total items correct at the immediate test were compared between children who returned and children who did not return through a series of t-tests. The two groups did not significantly differ on any of these variables.

Parents completed a questionnaire indicating whether they had returned to the building of training or seen the researcher from Experiment 1 during the delay. Six children had returned to the building, 1, 1, 1, 4, 4-5, and 5 times respectively. One child had returned to the building twice a month because her mother works there. A child who had returned to the building one time also saw the experimenter one time. A *t*-test revealed that children who did and did not return to the building did not differ in their performance on the very long-term test, *t*(17) = 0.027, *p* = 0.979, Cohen’s *d* = 0.013.

#### Stimuli

The object sets and forms that were used during initial training were used for the very long-term test.

#### Procedure

Children participated in Experiment 1 between September 2014 and May 2014, a range of 9 months. For the very long-term test, they were contacted and asked to return between September 2015 and December 2015. Thus, there were variations in how much time had passed since training for each child (range = 5 months and 26 days to 1 year and 24 days, mean 9 months and 22 days). Children who completed training earlier during Experiment 1 were likely to have a longer delay between training and the very long-term test than children who completed training later. We assessed whether the length of the delay was related to: mother’s education, father’s education, raw PPVT score, raw PLS comprehension and expression scores, and performance on the short-term dot test by running correlations. The correlation between the length of the delay and mother’s education approached significance *r* = 0.452, *p* = 0.052. The mothers of the children who had a longer delay had a higher level of education than mothers of children who had a shorter delay. However, mother’s education was not significantly correlated with children’s performance on the dot test at the very long-term test, *r* = 0.088, *p* = 0.720. None of the other correlations were significant.

Children were assigned to return to the room with the couch in the building where they completed initial training during week 1, or to come to a room with a child-sized table and two chairs in a building on the other side of campus. Children who returned to the familiar room saw the same researcher again while children who went to the new building met with a researcher they had never seen before. The assignment of the very long-term context was balanced as much as possible for age of the children during initial training (3-year-olds, 4- and 5-year-olds) and whether the child was a high or low scorer on the short-term dot test (based on a median split for each age group).

To ascertain children’s memory of the previous training context, the experimenter showed them the four photos of the room with the couch that varied in décor and furniture arrangement. They were asked to pick the room they had visited previously. For children returning to the same building, the experimenter administered this probe before the session began while still outside the closed room. In this case, the experimenter gestured toward the room and asked, “Do you remember when you came here before and looked at some things in that room? It was a long time ago. Do you remember what that room looked like? Pick which one it looked the most like. If you are not sure, you can just guess.” For children returning to a different building, the experimenter administered the probe after the session ended to prevent reactivating the memory of the training context before testing children’s memory for the forms. These children were asked, “Do you remember when you looked at these things before in a different room? It was a long time ago. Do you remember what that room looked like? Pick the one it looked the most like. If you are not sure, you can just guess.” Other than asking about the rooms, the procedure was identical for the two groups of children.

Children first completed the same practice trials (with a car and a book) that they completed during initial training to re-familiarize them with the dot test. They were then administered the dot test following the same procedure as the short-term delay (with the prototype exemplar of the object; the same target form, minimally different form, and maximally different form; and with the order of presentation the same). Children were tested on the set they were trained on first followed by the set they were trained on second.

After both object sets were tested, the child and the experimenter stood on the rug and completed 3 stretches (about 3 mins). Children were then retrained on the first set they were trained on during Experiment 1 following the exact same training protocol. To avoid fatigue, only the first set was retrained. Testing was then administered in the same way as the short-term test. The only difference was that the order in which the forms were presented (target, minimally different, maximally different form) was different from the order in the short-term test. The overall order (i.e., the 2nd, 4th, and 6th objects trained were tested first followed by the 1st 3rd, and 5th) remained the same across all tests.

### Results

A series of *t*-tests was conducted to compare the number of times children selected the target form to chance (4 correct responses out of 12 3AFC trials, Bonferroni Correction, 0.05/2 = 0.025). Children who completed the very long-term test in the familiar context performed significantly above chance, *t*(8) = 3.043, *p* = 0.016, Cohen’s *d* = 1.014 as did children who completed the test in the novel context, *t*(9) = 3.115, *p* = 0.012, Cohen’s *d* = 0.985. Children’s performance on the short-term, long-term, and very long-term tests were compared through a repeated measures ANOVA. This analysis revealed a significant effect for delay, *F*(2) = 7.08, *p* = 0.003, $\eta_{p}^{2}$ = 0.282. *Post hoc* analyses (Bonferroni correction, 0.05/3 = 0.016) revealed that children performed significantly worse after the very long-term delay than they did after the short-term *t*(18) = 3.076, *p* = 0.007, Cohen’s *d* = 0.723, or the long-term delay *t*(18) = 3.167, *p* = 0.005, Cohen’s *d* = 0.781. Recall that children’s performance at the short- and long-term delay did not differ, *t*(18) = 0.205, *p* = 0.840, Cohen’s *d* = 0.045.

To assess the effects of various factors on children’s memory for forms, a mixed effects logistic regression in an R environment, using the lme4 package, was conducted. The maximal random effects structure supported by the data included random intercepts for participant and item, and a random slope for participant over time. We systematically tested the same predictors as in the long-term test, with the addition of two measures: a time delay measure and accuracy for specific items at the long-term test. All very long-term models included a measure of time since the training as a covariate. Time delay from training was measured in days and standardized via conversion to group *z*-scores prior to entry in the models.

There were no reliable differences in performance associated with order of training (week 1, week 2) during the initial experiment, object set (A, B), or context of the very long-term test (novel, familiar) (all *p* > 0.10). Model fit did not drop when omitting these predictors. Thus, all data were collapsed across order, training set, and context for the remainder of the analyses.

**FIGURE 2:**
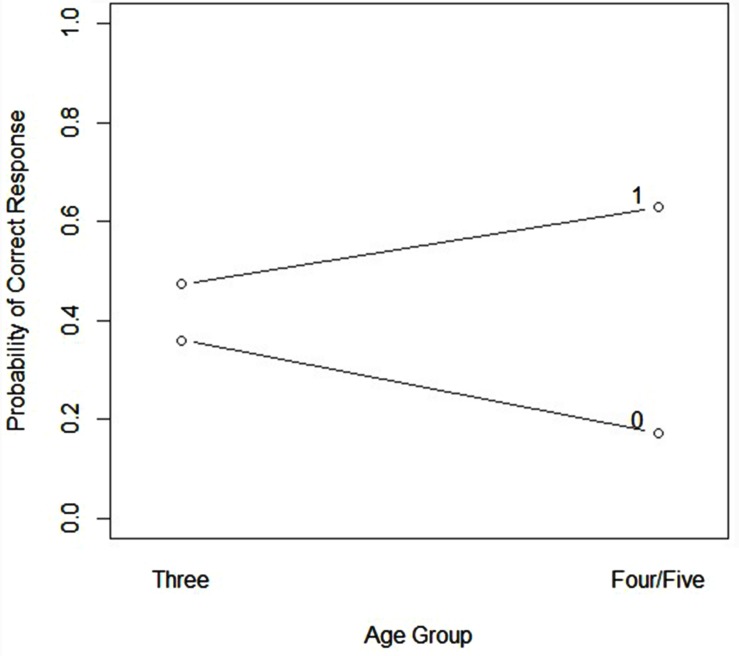
**Likelihood of correct response on very long-term test plotted by response accuracy on short-term test and age, short-term accuracy is coded as 0 (incorrect) or 1 (correct) above**.

The final model included main effects for response at the short-term test, age, and delay from training, as well as an interaction between response at the short-term test and age (See **Table 5**). A trend emerged for a negative association between time delay since training (standardized) and log odds of correct response (*z* = -1.92, *p* = 0.06). No significant main effects or interactions emerged for any of the language-based predictors. Inclusion of PPVT-IV or either of the PLS-4 scores did not improve model fit. Thus, these language measures were dropped from the final model. No reliable effect of child accuracy on the long-term test on log odds of a correct response at the very-long-term test was detected (*z* = 0.30). A significant interaction between age and short-term test response emerged (*z* = 1.99, *p* < 0.05). Three-year-olds tended to show little effect of prior accuracy at the short-term test on very long-term performance. Four- and 5-year-old participants demonstrated increased log odds of a correct response at very long-term in association with a correct short-term response, and decreased log odds of a correct response at very long-term in association with an incorrect short-term response. See **Figure 2**.

**Table 5:** Final model for very-long-term test.

	Estimate	Standard error	*z* value	Pr(>|z|)
Intercept	-0.50	0.33	-1.52	0.13
Short-term response^a^	0.48	0.37	1.31	0.19
Age^b^	-1.01	0.71	-1.42	0.16
Delay (Standardized)^c^	-0.29	0.15	-1.92	0.06
Age ^∗^ Short-term response	1.67	0.80	2.08	0.04


*^a^reference group is incorrect; ^b^reference group is 3-year-olds; ^c^delay in days from long-term test, standardized.*

#### Word-Level Factors

The number of target forms, minimally different forms, and maximally different forms that children selected at the very long-term test were compared through a series of *t*-tests (Bonferroni correction, 0.05/3 = 0.016). Children were more likely to select the target than the minimally different form, *t*(18) = 4.824, *p* < 0.0001, Cohen’s *d* = 2.291; and more likely to select the target than the maximally different form, *t*(18) = 3.343, *p* = 0.004, Cohen’s *d* = 1.952; but the number of times that children selected the minimally different or maximally different form did not differ significantly from each other, *t*(31) = 1.632, *p* = 0.120, Cohen’s *d* = 0.408.

In addition to assessing children’s responses to the target, a series of *t*-tests was conducted to assess whether children’s number of selections of the minimally different form and maximally different form differed at the short-term and very long-term test (Bonferroni correction, 0.05/3 = 0.016). Consistent with the results of the mixed effects logistic regression, children selected significantly more target forms in the short-term than the very long-term test, *t*(18) = 3.076, *p* = 0.007, Cohen’s *d* = 1.064. Children’s selections of the minimally different form did not differ across the two time points, *t*(18) = 0.942, *p* = 0.359, Cohen’s *d* = 0.259. However, children selected significantly more maximally different forms at the very long-term test than they did at the short-term test, *t*(18) = 3.311, *p* = 0.004, Cohen’s *d* = 0.760. While the number of maximally different forms increased between the short- and very long-term test, maximally different choices still did not differ significantly from chance at the very long-term test, *t*(18) = 1.606, *p* = 0.126, Cohen’s *d* = 0.368. Selections of the target remained significantly above chance, *t*(18) = 4.303, *p* < 0.0001, Cohen’s *d* = 0.987, while selections of the minimally different form were significantly below chance at the very long-term test, *t*(18) = 4.440, *p* < 0.0001, Cohen’s *d* = 1.019.

We analyzed whether children were more likely to select the minimally different form at the very long-term test based on the position of change (initial, medial, final) from the target. An ANOVA comparing number of near neighbor choices based on position of change revealed no significant effect for position of change, *F*(2,54) = 0.848, *p* = 0.434, $\eta_{p}^{2}$ = 0.030. We also compared children’s selection of the target form based on whether the form was a one-syllable or two-syllable word through a *t*-test. This analysis revealed no significant difference for number of syllables, *t*(18) = 1.489, *p* = 0.154, Cohen’s *d* = 0.354.

Correlations were conducted to assess whether children’s selection of the target, the minimally different form, and the maximally different form were related to the length of the very long-term delay. Consistent with the results of the mixed effects logistic regression, the longer the delay the less likely children were to select the target form, *r* = 0.484, *p* = 0.036. Additionally, the longer the delay, the more likely children were to select the maximally different form *r* = 0.681, *p* = 0.001. However, the length of the delay was not related to the likelihood that children would select the minimally different form *r* = 0.077, *p* = 0.754.

#### Memory for Context

Children’s selections of the photos were compared to chance through a series of *t*-tests (Bonferroni correction, 0.05/3 = 0.016). Children’s selections of the photos did not differ significantly from chance: same décor, same arrangement, *t*(18) = 1.901, *p* = 0.073, Cohen’s *d* = 0.436; same décor, but a different arrangement, *t*(18) = 1.470, *p* = 0.159, Cohen’s *d* = 0.337; different décor and a different arrangement, could not be calculated as no children selected this photo; and different décor and a different arrangement *t*(18) = 2.001, *p* = 0.061, Cohen’s *d* = 0.459. A chi-square analyses revealed no significant relationships between photo selection and the novel or familiar context χ(2) = 0.560, *p* = 0.756.

#### Performance after Retraining

Children’s performance on the set that was retrained was compared to their performance on that set at the short-term test through a paired samples *t*-test. This analysis revealed no significant difference between performance after initial training and performance after retraining, *t*(18) = 0.680, *p* = 0.505, Cohen’s *d* = 0.169.

### Summary of Results

Both younger and older children demonstrated a memory for forms after a delay of 6 months to 1 year. Children performed worse after the very long-term delay than they did either 10 mins or 2 to 3 days after training and the longer the delay, the worse children performed. Additionally, there was an interaction such that older children’s performance on specific items at the short-term delay was related to their performance on those items at the very long-term delay, but this was not the case with the younger children. Children selected the target form reliably more than the other two forms at the very long-term test, and the number of selections of the minimally different form and maximally different form did not differ. However, the length of the very long-term delay was related to decreased choices of the target form and increased choices of the maximally different form.

## Experiment 3

One of the limitations of Experiments 1 and 2 is that during the dot test, children are presented with one form they heard during training (i.e., the target form) and two forms that they have never heard before (i.e., the minimally different and maximally different forms). Thus, children could respond correctly because they recognize the form that goes with the specific object presented, reflecting successful retention of word learning, or they could respond correctly merely because they are able to identify the form they heard during training. To address this limitation, at the end of the very long-term testing session, children were administered a four-dot version of the test. In this test, they were presented with a trained object and a piece of paper with two large squares and two dots within each square. They were then given the target form for the object, a minimal pair of the target, another form that was presented with another object during training, and a minimal pair of that form. For example, they were shown the keenit and asked, “What is this one called? Is it a keenit (while pointing to the first dot in the square on the left) is it a keegit (while pointing to the second dot in the square on the left) is it a plune (while pointing to the first dot in the square on the right) or is it a pluve (while pointing to the second dot in the square on the right)?”

Children’s pattern of errors in this test should provide further information about children’s memories for forms. If they select the minimal pair of the target most often when making errors, this provides evidence that children retain a strong link between the form and the object, but that the phonological representation of the form is not specific enough to distinguish between the target form and its minimal pair. Conversely, if they select the alternate trained form most often when making errors, this provides evidence that children retain fairly specific representations of forms, but that the link between the object and the form is not strong enough to reliably select the correct trained form.

### Materials and Methods

#### Participants

Participants included all of the children who participated in Experiment 2.

#### Procedure

After retraining and testing with the 3-dot test, children had another 3-min break with stretches. The children were then told, “We are going to play a game that is a little different, so pay attention.” They completed two practice trials with the book and the car to familiarize them with the 4-dot test. For example, they were shown the book and asked, “Is this a book, a gook, a lar, or a car?”

They were tested on the retrained word-object pairs in the same order as the objects were presented in all other tests. Whether the target form appeared in the right square or the left square was counterbalanced across trials, but the target form and its minimal pair were always presented in the same square. Also, the two trained forms were paired such that one-syllable words were always presented with two-syllable words (with the exception of the practice trials) and the location of the change (initial, medial, and final) differed between the two trained forms presented. Additionally, the same form was never presented in two consecutive test trials either as the target or the alternate trained form.

### Results

**FIGURE 3:**
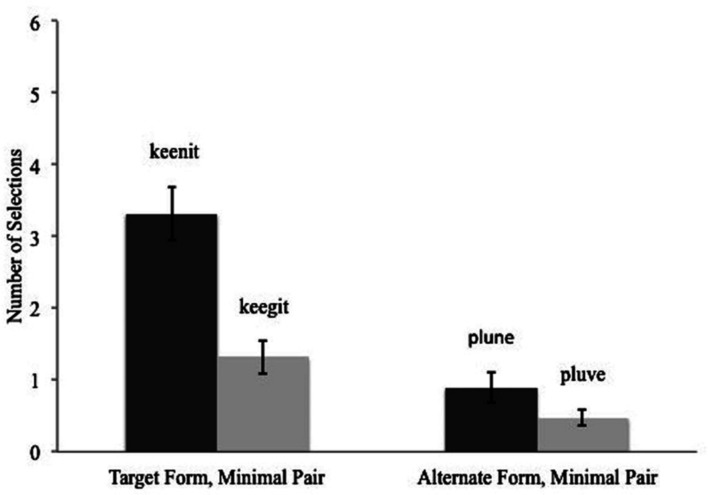
**Children’s responses in the 4-dot test.** Examples of a target form, an alternate trained form, and minimal pairs from a given trial are included in the figure.

Children’s responses on the 4-dot test were compared to chance (1.5 correct responses out of 6 4AFC trials) through a series of *t*-tests (Bonferroni correction, 0.05/4 = 0.0125). This analysis revealed that selections of the target form were significantly above chance, *t*(18) = 4.842, *p* < 0.0001, Cohen’s *d* = 1.111; selections of the minimal pair of the target *t*(18) = 0.849, *p* = 0.407, Cohen’s *d* = 0.195, and selections of the alternate trained form *t*(18) = 2.654, *p* = 0.016, Cohen’s *d* = 0.608, did not differ significantly from chance; and selections of the minimal pair of the alternate form were significantly below chance *t*(18) = 8.721, *p* < 0.0001, Cohen’s *d* = 2.001. A series of paired-samples *t*-tests (Bonferroni correction, 0.05/6 = 0.0008) revealed that children selected the target form significantly more than all other forms: the minimal pair of the target *t*(18) = 3.736, *p* = 0.002, Cohen’s *d* = 1.75; the alternate trained form *t*(18) = 4.219, *p* = 0.001, Cohen’s *d* = 2.93; and minimal pair of the alternate form *t*(18) = 6.349, *p* < 0.0001, Cohen’s *d* = 2.701. Also, there was a significant difference between children’s selection of the minimal pair of the target and the minimal pair of the alternate form *t*(18) = 3.281, *p* = 0.004, Cohen’s *d* = 0.859 (see **Figure 3**). There were no other significant differences.

### Summary of Results

When presented with four choices on the dot test, children were most likely to select the target form, revealing that they are able to recognize the form that was linked to a specific object during training. Children’s selected the other forms a similar number of times. Yet, their responses suggest that when in error, they are not responding completely at chance. Rather, their pattern of responses show both a sensitivity to which forms they heard during training (i.e., the target and the alternate trained form) and a sensitivity to which forms are phonetically similar to the form that is linked to the target object (i.e., the target and its minimal pair).

## Discussion

The primary questions of the current study were: (1) How does the number of word forms that children correctly identify change based on the length of the delay?, and (2) How do children’s phonological representations of word forms change based on the length of the delay? With regards to the first question, after a minimal number of exposures during training children retrieved the correct form above chance at all time delays (10 mins, 2 to 3 days, and 6 months to 1 year after training) when they were given three forms to choose from and were allowed to respond manually instead of verbally. Critically, the dot test was sensitive enough to show that after an extended delay a memory of the forms remain. Additionally, not only 4- and 5-year-old children, but also 3-year-old children were able to demonstrate a reliable memory of forms through the dot test, even after an extended delay.

Although children reliably demonstrated a memory for word forms after the extended delay, we did see a decrement to their memory such that they correctly identified fewer forms 6 months to 1 year after training than they did 10 mins or 2 to 3 days after training. Additionally, their very long-term performance was related to the length of the delay in that children who were tested after a shorter delay did better than children who were tested after a longer delay. Thus, although the dot test included reduced task demands that allowed children to show memory for the forms after a very long-term delay, the test remained sensitive enough to show changes based on the length of that delay. This finding suggests that even with good encoding, memory continues to decline over longer intervals without further exposures to word-referent pairs, but that the rate of forgetting can be very slow.

The current findings stand in contrast to traditional assessments of word forms in which children are asked to recall and produce forms and perform near floor immediately after testing. Clearly, the reduced task demands of the dot test allowed children to demonstrate a memory for forms both over shorter and longer post-training delays. However, how do we reconcile the current findings with work such as Horst and Samuelson (2008), in which children responded at chance just 5 mins after training in a referent task that allowed for recognition memory and manual responses? This research varies from the current work in a number of critical ways. Namely, in Horst and Samuelson children were 2-years-old, they only heard each novel word once during training, and they learned the novel words through a referent selection task instead of an explicit naming task. Overall, past and current research point to key factors that affect children’s ability to retrieve trained words including: the child’s age, the type of training they receive, and the task demands at retrieval. Given the relative scarcity of research investigating retention of words across various post-training delays, more research is needed in which we systematically vary these factors to gain a better understanding of how they affect retention (see Vlach and Sandhofer, 2012 for an example of this type of work). With regards to follow-up work for the current study, it would be informative to investigate how the type of assessment (e.g., referent vs. form tests) and the type of memory supports given during retrieval (e.g., free recall, cued recall, recognition tests) affect children’s ability to retrieve word-referent pairs at various delay intervals. Our previous work (Gordon and McGregor, 2014) suggests that even when the task demands are made to be more comparable, preschool-age children perform better on referent tests than form tests after a week’s delay. Yet, it is currently unknown whether performance in these two tests would differ after longer retention intervals. Additionally, several days to 6 months after training is a large gap in time. Thus, additional research in which we further vary the length of the delay would provide a more detailed understanding of how memories for forms change after encoding.

With regards to the second question, children were no more likely to select the minimally different form than the maximally different form on the 3-dot test at all time points. These results suggest that children encode and maintain fairly specific representations of forms, and that over time the form is more likely to be forgotten than it is to be remembered in a less phonologically precise way. One limitation to our current methodology is that children were presented with the same minimal pair for each target form across all tests. Thus, it is possible that children encode fuzzy phonological representations of forms, or that the representations became more imprecise over time, but these representations were not captured by the variation between minimal pairs and targets in the current study. Further research will be needed to clarify whether this was the case.

Results from the 4-dot test administered 10 mins after training do not address the question of how phonological representations change across various post-training delays. However, these results do offer some insights into how children weigh their memory for the specific phonological forms that they heard during training and their memory for word-object links. Specifically, when given the target form for the object presented, another trained form, and minimal pairs of both children were most likely to select the target form, demonstrating good memory for both the form and the word-referent link. Interestingly, the number of times children selected the other 3 forms show a graded response. Namely, when in error they were most likely to select the minimal pair of the target, and least likely to select the minimal pair of the alternate trained form. These results suggest that children are sensitive to tracking information about word-object links and the specific phonemes of forms presented during training and retain this information over short retention intervals. Further research can reveal how children’s memory for both links and forms changes across various delays.

In addition to these primary questions, we explored whether individual factors (i.e., age, vocabulary level, and general language abilities) and environmental factors (i.e., location and décor of the room, and the experimenter administering the training and test) were related to children’s retention of words. With regards to age, older children were better at encoding and retaining words across short delays than younger children, but differences due to age disappeared at the very long-term test. Few researchers have investigated age differences in the retention of novel words over longer delays. One exception is Vlach and Sandhofer (2012) who found a difference between the number of adults and the number of 3-year-old children who maintained a word-referent mapping over short-term delays (i.e., immediate, 1 week), but found no difference between these groups after a month’s delay. These findings as well as the findings in the current paper suggest that age differences in memory for words are minimized over time without additional exposures to those words.

Although, older and younger children did not show differences in the number of correct responses at the very long-term test, there were differences in their responses to specific items. Namely, 4- and 5-year-old children’s correct response to a specific item at the short-term test was related to their correct response to that item at the long-term and very long-term tests. The same was not true for the 3-year-olds. There could be a variety of reasons for this difference. Younger children could have formed a weaker memory trace for each item than older children, which led to more variable responses. Another possibility is that the memory trace for each word-object link was similarly strong for both age groups, but that the older children were better at retrieving links than younger children after a delay. Although, this is possible, Brainerd et al.’s (1990) work provides evidence that children’s ability to encode and retain information improves across development while their ability to retrieve information remains fairly stable. Recent research provides support for this interpretation. Namely, a wide variety of studies on event memory provide evidence that the amount and quality of information encoded and the length of time children retain a memory gradually improves across development (see Bauer, 2015a,b for reviews). Research investigating the relationship between neural development and memory development in infancy also support these behavioral findings (Nelson, 1995; Richmond and Nelson, 2007). Despite evidence for these developmental trends, we lack a good understanding of how encoding and retention of words, and other semantic information, changes during the preschool years. However, there is some research that supports the same conclusions. Bauer et al. (2012) found that 4-year-old children demonstrated a better memory for the order of multi-step events than 3-year-olds across delay intervals (i.e., immediately after training, 1 week, and 1 month post-training), demonstrating developmental differences in retention. However, all children retrieved more information when given more support during testing, such as verbal cues, but there were no age differences based on level of support given during testing. This finding suggests no developmental changes in children’s ability to retrieve information.

In addition to the effect of age on encoding and retention of forms, current language abilities are likely to explain individual differences as well. For example, there is evidence that the more words children learn, the more specific their phonological representations become and the better they are at encoding novel words (Metsala and Walley, 1998; Werker and Tees, 1999). Additionally, more experience producing speech sounds fosters better encoding and production of those speech sounds (Keren-Portnoy et al., 2010). However, the majority of this work is conducted with infants. Within the current study, we found no evidence of a relationship between vocabulary and children’s word learning ability, as tested at the 10-min delay, or their retention of words after the very long-term delay. However, we did find a relationship between vocabulary and retention of newly trained forms after a short-delay, 2 to 3 days after training. The effect we found after the short delay was not very large (*z* = 2.04, *p* < 0.04). Therefore, the lack of a relationship at the longer delay may have been due to a lack of power and a trend toward floor effects. Critically, these results suggest that vocabulary comprehension is related to preschool-age children’s ability to maintain forms, at least across a delay of several days.

With regards to the environmental factors, children reliably encoded and retained information about the décor and arrangement of elements in the training room after the 2- to 3-day delay. However, they did not demonstrate a memory for the training room after a delay of 6 months to 1 year. Regardless, we did not find evidence of a context effect at any of the testing sessions. Context effects have been shown to be stronger for longer delays, yet effects are also more likely to be shown with more demanding memory tests (i.e., recall tests) as opposed to easier tests (i.e., recognition tests) (Smith and Vela, 2001). Thus, in the current study, either children did not link the context cues to the target information, or the tests were not sufficiently difficult for context cues to make a difference in performance. It is difficult to answer this question given that there is rapid development of children’s ability to bind elements of episodes between the ages of 4 and 6, and evidence of binding varies based on the type of learning and task difficulty (see Newcombe et al., 2012). Furthermore, because context effects tend to be relatively small (Smith and Vela, 2001) we could have failed to find context effects because of a lack of power. This seems unlikely as inspection of the data revealed no trend of children in familiar contexts performing better than children in novel contexts. The good news is that our results suggest that preschool-age children are good at generalizing word learning to a variety of environmental contexts and with a variety of adults.

Overall, current findings suggest that: (1) Children retain word forms that are linked to objects after a minimal number of exposures and a delay longer than 6 months, and (2) Children maintain phonologically specific representations of word forms over short and long delays. Additionally, the current work provides evidence that memories for words that are maintained over several days are not maintained indefinitely. Rather, memories for forms decrease slowly over longer time scales without further exposures. Overall, understanding word learning requires understanding how: age, current language abilities, type of training, type of test at retrieval (i.e., referent test, form test), amount of memory support given during retrieval (i.e., free recall, cued recall, recognition), and length of delay affects children’s ability to retrieve words. It is undoubtedly challenging to understand how the complex interaction of these factors lead to successful or unsuccessful retrieval of words. However, it is important that we continue to refine our understanding of the influence of these factors as it is through this understanding that we can maximize word learning in educational, clinical, and home environments.

## Author Contributions

KG conceived and carried out the research design and wrote the manuscript. KM, RG, and LS provided feedback both on the study design and the manuscript. BW helped design and carry out Experiments 2 and 3. MC ran all of the mixed effects logistic regressions.

## Conflict of Interest Statement

The authors declare that the research was conducted in the absence of any commercial or financial relationships that could be construed as a potential conflict of interest.
